# Dichlorido[(*S*)-*N*-(1-phenyl­ethyl­idene)-1-(pyridin-2-yl)ethanamine-κ^2^ *N*,*N*′]zinc(II) dichloro­methane solvate

**DOI:** 10.1107/S1600536810029387

**Published:** 2010-07-31

**Authors:** Saira Nayab, Seung-Min Paek, Jong Hwa Jeong

**Affiliations:** a Department of Chemistry, Kyungpook National University, Taegu, 702-701, Republic of Korea

## Abstract

In the title compound, [ZnCl_2_(C_15_H_16_N_2_)]·CH_2_Cl_2_, the Zn(II) atom has a distorted tetra­hedral coordination by two Cl atoms and two N atoms from the organic ligand [the average Zn—N and Zn—Cl bond lengths are 2.060 (4) Å and Zn—Cl = 2.179 (16) Å, respectively]. The dihedral angle between the N—Zn—N and Cl—Zn—Cl planes is 89.9 (1)°. The phenyl ring forms a dihedral angle of 40.6 (5)° with the imine plane.

## Related literature

For related structures see: Brunner & Fisch (1987 ▶); Nguyen & Jeong (2008 ▶).
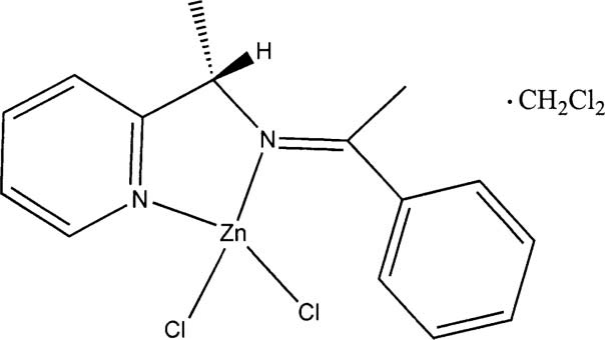


## Experimental

### 

#### Crystal data

[ZnCl_2_(C_15_H_16_N_2_)]·CH_2_Cl_2_
*M* *_r_* = 445.51
Triclinic, 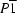

*a* = 7.9381 (7) Å
*b* = 10.7187 (8) Å
*c* = 11.8426 (7) Å
α = 96.724 (6)°
β = 108.466 (6)°
γ = 97.968 (7)°
*V* = 932.53 (13) Å^3^
*Z* = 2
Mo *K*α radiation
μ = 1.89 mm^−1^
*T* = 295 K
0.45 × 0.40 × 0.35 mm

#### Data collection

Enraf–Nonius CAD-4 diffractometer
Absorption correction: ψ scan (*ABSCALC*; McArdle & Daly, 1999 ▶) *T*_min_ = 0.489, *T*_max_ = 0.569
3871 measured reflections
3463 independent reflections
2873 reflections with *I* > 2σ(*I*)
*R*_int_ = 0.009
3 standard reflections every 60 min  intensity decay: none

#### Refinement

*R*[*F* ^2^ > 2σ(*F* ^2^)] = 0.059
*wR*(*F* ^2^) = 0.197
*S* = 1.11
3463 reflections
210 parameters
H-atom parameters constrained
Δρ_max_ = 0.85 e Å^−3^
Δρ_min_ = −1.38 e Å^−3^

### 

Data collection: *CAD-4 Software* (Enraf–Nonius, 1989 ▶); cell refinement: *CAD-4 Software*; data reduction: *XCAD* (McArdle, 1999 ▶); program(s) used to solve structure: *SHELXS97* (Sheldrick, 2008 ▶); program(s) used to refine structure: *SHELXL97* (Sheldrick, 2008 ▶); molecular graphics: *ORTEPIII* (Burnett & Johnson, 1996 ▶); software used to prepare material for publication: *SHELXL97*.

## Supplementary Material

Crystal structure: contains datablocks global, I. DOI: 10.1107/S1600536810029387/rk2219sup1.cif

Structure factors: contains datablocks I. DOI: 10.1107/S1600536810029387/rk2219Isup2.hkl

Additional supplementary materials:  crystallographic information; 3D view; checkCIF report

## Figures and Tables

**Fig. 1.**
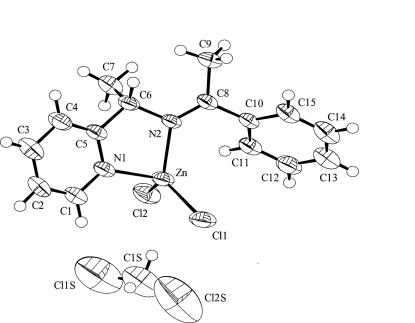
The molecular structure of the title compound with the atom numbering scheme. Displacement ellipsoids are drawn at the 40% probability level. H atoms are presented as a small spheres of arbitrary radius.

## supplementary crystallographic information

### Comment

The ligand, (*S*)–*N*–(1–phenylethylidene)–1–(pyridin–2–yl)ethanamine, was obtained from reaction of (*S*)–1–(pyridin–2–yl)ethanamine with acetophenone in toluene solution. The similar ligand was used in Rh or Zn complex (Brunner & Fisch, 1987; Nguyen & Jeong, 2008). The geometry around the Zn atom is nearly tetrahedral with two chlorine atoms and the one pyridyl and one amine nitrogen atoms of the ligand (Fig. 1). The dihedral angle between N—Zn—N and Cl—Zn—Cl planes is 89.9 (1)°. The phenyl cycle forms dihedral angle 40.6 (5)° with imine plane. This value may be explained by steric hinderance between the Cl atoms and phenyl moiety of organic ligand.

### Experimental

Acetophenone (2.04 g, 0.017 mol) was added to a solution of (*S*)–1–(pyridin–2–yl)ethanamine (2.07 g, 0.017 mol) in 40 ml of toluene. The reaction mixture was heated under reflux for 2 days in a Dean–Stark equipment. The solvent was removed by evaporation to yield pale brown oil, 3.43 g (90% yield). ^1^H NMR (400 MHz, CDCl_3_) δ 8.61 (t, 2H, *Ar*H), 8.28 (d, 2H, *Ar*H), 7.70 (m, 2H, *Ar*H), 7.24 (m, 2H, *Ar*H), 7.10 (m, 1H, *Ar*H), 5.04 (q, J = 6.56 Hz, 1H, CH), 2.42 (s, 3H, CH_3_), 1.44 (d, J = 6.56 Hz, 3H, CH_3_). A solution of the ligand (1.01 g, 4.5 mmol) in ethanol (5 ml) was added dropwise to a solution of ZnCl_2_ (0.61 g, 4.5 mmol) in ethanol (10 ml). The mixture was stirred overnight at room temperature. The solvent was removed to yield white solid product. Colourless crystals were obtained by slow difussion of hexane to CH_2_Cl_2_ solution (1.43 g, 80%). Anal. Cacld. For [C_15_H_16_Cl_2_N_2_Zn]^.^CH_2_Cl_2_: C, 43.14%; H, 4.07%; N, 6.29%. Found: C, 43.04%; H, 4.12%, N, 6.19%. ^1^H NMR (400 MHz, CDCl_3_) δ 8.63 (t, 2H, *Ar*H), 8.44 (d, 2H, *Ar*H), 7.81 (m, 2H, *Ar*H), 7.24 (m, 2H, *Ar*H), 7.11 (m, 1H, *Ar*H), 5.23 (m, 1H, CH), 2.56 (s, 3H, CH_3_), 2.00 (d, J = 6.42 Hz, 3H, CH_3_).

### Refinement

All H atoms were positioned geometrically and refined using a riding model with 0.96Å and *U*_iso_(H) = 1.5*U*_eq_(C) for CH_3_ groups; 0.93Å for aryl H atoms, 0.97Å for mehylene H atoms and 0.98Å for methine H atom with *U*_iso_(H) = 1.2*U*_eq_(C). The maximum remaining electron density was 0.85e/Å^3^ at 0.802Å from Zn.

### Crystal data

**Table:**

[ZnCl_2_(C_15_H_16_N_2_)]·CH_2_Cl_2_	*Z* = 2
*M**_r_* = 445.51	*F*(000) = 452
Triclinic, *P*1	*D*_x_ = 1.587 Mg m^−^^3^
Hall symbol: -P 1	Mo *K*α radiation, λ = 0.71073 Å
*a* = 7.9381 (7) Å	Cell parameters from 25 reflections
*b* = 10.7187 (8) Å	θ = 10–12°
*c* = 11.8426 (7) Å	µ = 1.89 mm^−^^1^
α = 96.724 (6)°	*T* = 295 K
β = 108.466 (6)°	Block, colourless
γ = 97.968 (7)°	0.45 × 0.40 × 0.35 mm
*V* = 932.53 (13) Å^3^	

### Data collection

**Table:**

Enraf–Nonius CAD-4 diffractometer	2873 reflections with *I* > 2σ(*I*)
Radiation source: fine–focus sealed tube	*R*_int_ = 0.009
graphite	θ_max_ = 25.5°, θ_min_ = 1.8°
ω/2θ–scans	*h* = 0→9
Absorption correction: ψ scan (*ABSCALC*; McArdle & Daly, 1999)	*k* = −12→12
*T*_min_ = 0.489, *T*_max_ = 0.569	*l* = −14→13
3871 measured reflections	3 standard reflections every 60 min
3463 independent reflections	intensity decay: none

### Refinement

**Table:**

Refinement on *F*^2^	Primary atom site location: structure-invariant direct methods
Least-squares matrix: full	Secondary atom site location: difference Fourier map
*R*[*F*^2^ > 2σ(*F*^2^)] = 0.059	Hydrogen site location: inferred from neighbouring sites
*wR*(*F*^2^) = 0.197	H-atom parameters constrained
*S* = 1.11	*w* = 1/[σ^2^(*F*_o_^2^) + (0.124*P*)^2^ + 1.3285*P*] where *P* = (*F*_o_^2^ + 2*F*_c_^2^)/3
3463 reflections	(Δ/σ)_max_ = 0.001
210 parameters	Δρ_max_ = 0.85 e Å^−^^3^
0 restraints	Δρ_min_ = −1.38 e Å^−^^3^

### Special details

**Table:**

Geometry. All s.u.'s (except the s.u. in the dihedral angle between two l.s. planes) are estimated using the full covariance matrix. The cell s.u.'s are taken into account individually in the estimation of s.u.'s in distances, angles and torsion angles; correlations between s.u.'s in cell parameters are only used when they are defined by crystal symmetry. An approximate (isotropic) treatment of cell s.u.'s is used for estimating s.u.'s involving l.s. planes.

### Fractional atomic coordinates and isotropic or equivalent isotropic displacement parameters (Å^2^)

**Table:**

	*x*	*y*	*z*	*U*_iso_*/*U*_eq_	
Zn	0.75251 (7)	0.65883 (5)	0.79296 (5)	0.0462 (2)	
Cl1	0.57114 (18)	0.80013 (15)	0.78335 (15)	0.0610 (4)	
Cl2	0.6555 (2)	0.45749 (14)	0.80264 (16)	0.0704 (5)	
N1	0.8371 (6)	0.6589 (4)	0.6459 (4)	0.0460 (9)	
N2	1.0239 (5)	0.7302 (4)	0.8835 (3)	0.0385 (8)	
C1	0.7332 (8)	0.6417 (6)	0.5294 (5)	0.0572 (13)	
H1	0.6083	0.6236	0.5100	0.069*	
C2	0.8008 (9)	0.6494 (6)	0.4393 (5)	0.0631 (15)	
H2	0.7244	0.6359	0.3594	0.076*	
C3	0.9837 (10)	0.6773 (7)	0.4673 (6)	0.0697 (17)	
H3	1.0336	0.6839	0.4065	0.084*	
C4	1.0944 (8)	0.6959 (6)	0.5869 (5)	0.0578 (14)	
H4	1.2195	0.7151	0.6077	0.069*	
C5	1.0162 (7)	0.6852 (5)	0.6742 (4)	0.0428 (10)	
C6	1.1306 (6)	0.6999 (5)	0.8060 (4)	0.0415 (10)	
H6	1.2345	0.7695	0.8246	0.050*	
C7	1.1975 (8)	0.5778 (6)	0.8317 (5)	0.0587 (14)	
H7A	1.2645	0.5867	0.9163	0.088*	
H7B	1.2744	0.5605	0.7857	0.088*	
H7C	1.0963	0.5085	0.8094	0.088*	
C8	1.1009 (6)	0.7966 (5)	0.9897 (4)	0.0410 (10)	
C9	1.2985 (7)	0.8533 (6)	1.0420 (5)	0.0597 (14)	
H9A	1.3577	0.8262	0.9868	0.090*	
H9B	1.3503	0.8252	1.1174	0.090*	
H9C	1.3141	0.9449	1.0554	0.090*	
C10	0.9907 (6)	0.8173 (5)	1.0667 (4)	0.0406 (10)	
C11	0.8521 (7)	0.7222 (5)	1.0654 (5)	0.0516 (12)	
H11	0.8315	0.6425	1.0178	0.062*	
C12	0.7449 (8)	0.7450 (7)	1.1340 (6)	0.0623 (15)	
H12	0.6519	0.6805	1.1317	0.075*	
C13	0.7731 (9)	0.8608 (7)	1.2053 (6)	0.0654 (16)	
H13	0.6987	0.8753	1.2504	0.078*	
C14	0.9100 (10)	0.9546 (6)	1.2103 (6)	0.0665 (16)	
H14	0.9294	1.0331	1.2595	0.080*	
C15	1.0214 (8)	0.9348 (6)	1.1429 (5)	0.0557 (13)	
H15	1.1163	0.9993	1.1481	0.067*	
Cl1S	0.4042 (8)	0.8397 (6)	0.3886 (5)	0.204 (2)	
Cl2S	0.2730 (12)	1.0098 (6)	0.5042 (6)	0.243 (3)	
C1S	0.368 (2)	0.9595 (16)	0.4503 (14)	0.168 (7)	
H1S1	0.4877	0.9926	0.5093	0.201*	
H1S2	0.3596	1.0119	0.3880	0.201*	

### Atomic displacement parameters (Å^2^)

**Table:**

	*U*^11^	*U*^22^	*U*^33^	*U*^12^	*U*^13^	*U*^23^
Zn	0.0334 (3)	0.0516 (4)	0.0525 (4)	0.0000 (2)	0.0164 (3)	0.0086 (3)
Cl1	0.0397 (7)	0.0689 (9)	0.0770 (9)	0.0123 (6)	0.0210 (6)	0.0166 (7)
Cl2	0.0694 (10)	0.0540 (8)	0.0794 (10)	−0.0110 (7)	0.0235 (8)	0.0106 (7)
N1	0.040 (2)	0.053 (2)	0.043 (2)	0.0033 (17)	0.0138 (17)	0.0084 (18)
N2	0.0327 (19)	0.044 (2)	0.0399 (19)	0.0044 (15)	0.0150 (16)	0.0062 (16)
C1	0.050 (3)	0.065 (3)	0.048 (3)	0.005 (2)	0.008 (2)	0.005 (2)
C2	0.070 (4)	0.072 (4)	0.040 (3)	0.007 (3)	0.011 (3)	0.006 (3)
C3	0.083 (4)	0.085 (4)	0.048 (3)	0.011 (3)	0.033 (3)	0.013 (3)
C4	0.054 (3)	0.073 (4)	0.050 (3)	0.008 (3)	0.026 (3)	0.007 (3)
C5	0.042 (2)	0.042 (2)	0.044 (2)	0.0045 (19)	0.015 (2)	0.0058 (19)
C6	0.033 (2)	0.051 (3)	0.043 (2)	0.0041 (19)	0.0182 (19)	0.006 (2)
C7	0.060 (3)	0.069 (4)	0.054 (3)	0.028 (3)	0.022 (3)	0.011 (3)
C8	0.040 (2)	0.042 (2)	0.041 (2)	0.0069 (19)	0.0132 (19)	0.0090 (19)
C9	0.041 (3)	0.074 (4)	0.055 (3)	0.002 (3)	0.013 (2)	−0.004 (3)
C10	0.040 (2)	0.046 (2)	0.038 (2)	0.0100 (19)	0.0141 (19)	0.0112 (19)
C11	0.051 (3)	0.055 (3)	0.051 (3)	0.004 (2)	0.020 (2)	0.014 (2)
C12	0.055 (3)	0.079 (4)	0.062 (3)	0.009 (3)	0.029 (3)	0.025 (3)
C13	0.074 (4)	0.084 (4)	0.060 (3)	0.034 (3)	0.040 (3)	0.025 (3)
C14	0.087 (5)	0.064 (4)	0.057 (3)	0.024 (3)	0.033 (3)	0.004 (3)
C15	0.062 (3)	0.053 (3)	0.051 (3)	0.008 (3)	0.022 (3)	0.004 (2)
Cl1S	0.228 (5)	0.247 (6)	0.205 (5)	0.112 (5)	0.120 (4)	0.085 (4)
Cl2S	0.366 (10)	0.203 (5)	0.186 (5)	0.083 (6)	0.120 (6)	0.022 (4)
C1S	0.155 (12)	0.176 (14)	0.126 (10)	0.047 (11)	0.004 (9)	−0.051 (10)

### Geometric parameters (Å, °)

**Table:**

Zn—N1	2.055 (4)	C7—H7C	0.9600
Zn—N2	2.065 (4)	C8—C10	1.469 (7)
Zn—Cl2	2.2175 (16)	C8—C9	1.499 (7)
Zn—Cl1	2.2182 (15)	C9—H9A	0.9600
N1—C5	1.333 (6)	C9—H9B	0.9600
N1—C1	1.340 (7)	C9—H9C	0.9600
N2—C8	1.282 (6)	C10—C11	1.388 (7)
N2—C6	1.471 (6)	C10—C15	1.403 (7)
C1—C2	1.343 (9)	C11—C12	1.375 (8)
C1—H1	0.9300	C11—H11	0.9300
C2—C3	1.363 (10)	C12—C13	1.365 (9)
C2—H2	0.9300	C12—H12	0.9300
C3—C4	1.384 (9)	C13—C14	1.355 (10)
C3—H3	0.9300	C13—H13	0.9300
C4—C5	1.372 (7)	C14—C15	1.388 (8)
C4—H4	0.9300	C14—H14	0.9300
C5—C6	1.513 (7)	C15—H15	0.9300
C6—C7	1.507 (8)	Cl1S—C1S	1.509 (14)
C6—H6	0.9800	Cl2S—C1S	1.268 (15)
C7—H7A	0.9600	C1S—H1S1	0.9700
C7—H7B	0.9600	C1S—H1S2	0.9700
			
N1—Zn—N2	81.55 (16)	C6—C7—H7C	109.5
N1—Zn—Cl2	107.97 (13)	H7A—C7—H7C	109.5
N2—Zn—Cl2	116.13 (12)	H7B—C7—H7C	109.5
N1—Zn—Cl1	107.49 (13)	N2—C8—C10	118.5 (4)
N2—Zn—Cl1	115.59 (12)	N2—C8—C9	124.1 (4)
Cl2—Zn—Cl1	120.09 (6)	C10—C8—C9	117.3 (4)
C5—N1—C1	118.7 (5)	C8—C9—H9A	109.5
C5—N1—Zn	114.0 (3)	C8—C9—H9B	109.5
C1—N1—Zn	127.2 (4)	H9A—C9—H9B	109.5
C8—N2—C6	120.5 (4)	C8—C9—H9C	109.5
C8—N2—Zn	129.0 (3)	H9A—C9—H9C	109.5
C6—N2—Zn	110.4 (3)	H9B—C9—H9C	109.5
N1—C1—C2	123.1 (6)	C11—C10—C15	118.0 (5)
N1—C1—H1	118.5	C11—C10—C8	121.2 (5)
C2—C1—H1	118.5	C15—C10—C8	120.8 (5)
C1—C2—C3	118.7 (5)	C12—C11—C10	120.5 (5)
C1—C2—H2	120.6	C12—C11—H11	119.8
C3—C2—H2	120.6	C10—C11—H11	119.8
C2—C3—C4	119.5 (6)	C13—C12—C11	121.0 (6)
C2—C3—H3	120.3	C13—C12—H12	119.5
C4—C3—H3	120.3	C11—C12—H12	119.5
C5—C4—C3	118.7 (6)	C14—C13—C12	119.8 (6)
C5—C4—H4	120.6	C14—C13—H13	120.1
C3—C4—H4	120.6	C12—C13—H13	120.1
N1—C5—C4	121.3 (5)	C13—C14—C15	120.8 (6)
N1—C5—C6	117.6 (4)	C13—C14—H14	119.6
C4—C5—C6	121.1 (5)	C15—C14—H14	119.6
N2—C6—C7	108.9 (4)	C14—C15—C10	119.9 (6)
N2—C6—C5	110.2 (4)	C14—C15—H15	120.1
C7—C6—C5	110.2 (4)	C10—C15—H15	120.1
N2—C6—H6	109.2	Cl2S—C1S—Cl1S	147.9 (16)
C7—C6—H6	109.2	Cl2S—C1S—H1S1	99.8
C5—C6—H6	109.2	Cl1S—C1S—H1S1	99.8
C6—C7—H7A	109.5	Cl2S—C1S—H1S2	99.8
C6—C7—H7B	109.5	Cl1S—C1S—H1S2	99.8
H7A—C7—H7B	109.5	H1S1—C1S—H1S2	104.1
			
N2—Zn—N1—C5	8.3 (3)	Zn—N2—C6—C7	−94.2 (4)
Cl2—Zn—N1—C5	−106.5 (3)	C8—N2—C6—C5	−150.7 (4)
Cl1—Zn—N1—C5	122.6 (3)	Zn—N2—C6—C5	26.8 (4)
N2—Zn—N1—C1	−168.3 (5)	N1—C5—C6—N2	−21.5 (6)
Cl2—Zn—N1—C1	76.9 (5)	C4—C5—C6—N2	159.9 (5)
Cl1—Zn—N1—C1	−54.0 (5)	N1—C5—C6—C7	98.6 (5)
N1—Zn—N2—C8	157.6 (4)	C4—C5—C6—C7	−79.9 (6)
Cl2—Zn—N2—C8	−96.5 (4)	C6—N2—C8—C10	−176.1 (4)
Cl1—Zn—N2—C8	52.2 (4)	Zn—N2—C8—C10	6.9 (7)
N1—Zn—N2—C6	−19.7 (3)	C6—N2—C8—C9	2.8 (7)
Cl2—Zn—N2—C6	86.2 (3)	Zn—N2—C8—C9	−174.2 (4)
Cl1—Zn—N2—C6	−125.1 (3)	N2—C8—C10—C11	36.8 (7)
C5—N1—C1—C2	0.2 (9)	C9—C8—C10—C11	−142.2 (5)
Zn—N1—C1—C2	176.7 (5)	N2—C8—C10—C15	−142.5 (5)
N1—C1—C2—C3	−0.8 (10)	C9—C8—C10—C15	38.5 (7)
C1—C2—C3—C4	0.6 (11)	C15—C10—C11—C12	2.2 (8)
C2—C3—C4—C5	0.1 (10)	C8—C10—C11—C12	−177.1 (5)
C1—N1—C5—C4	0.5 (8)	C10—C11—C12—C13	−0.5 (9)
Zn—N1—C5—C4	−176.4 (4)	C11—C12—C13—C14	−0.9 (9)
C1—N1—C5—C6	−178.0 (5)	C12—C13—C14—C15	0.5 (10)
Zn—N1—C5—C6	5.0 (6)	C13—C14—C15—C10	1.2 (9)
C3—C4—C5—N1	−0.7 (9)	C11—C10—C15—C14	−2.5 (8)
C3—C4—C5—C6	177.9 (5)	C8—C10—C15—C14	176.8 (5)
C8—N2—C6—C7	88.3 (5)		
